# Senecavirus A seroprevalence and risk factors in United States pig farms

**DOI:** 10.3389/fvets.2022.1011975

**Published:** 2022-10-20

**Authors:** Guilherme Preis, Juan M. Sanhueza, Carles Vilalta, Fabio A. Vannucci, Marie R. Culhane, Cesar A. Corzo

**Affiliations:** ^1^Veterinary Population Medicine Department, College of Veterinary Medicine, University of Minnesota, Saint Paul, MN, United States; ^2^Departamento de Ciencias Veterinarias y Salud Pública, Facultad de Recursos Naturales, Universidad Católica de Temuco, Temuco, Chile; ^3^Unitat Mixta d'Investigació IRTA-UAB en Sanitat Animal, Centre de Recerca en Sanitat Animal, Campus de la Universitat Autònoma de Barcelona, Bellaterra, Catalonia, Spain; ^4^IRTA, Programa de Sanitat Animal, Centre de Recerca en Sanitat Animal, Campus de la Universitat Autònoma de Barcelona, Bellaterra, Catalonia, Spain; ^5^University of Minnesota Veterinary Diagnostic Laboratory, College of Veterinary Medicine, University of Minnesota, St. Paul, MN, United States

**Keywords:** senecavirus A, seroprevalence, risk factors, vesicular disease, swine

## Abstract

Senecavirus A (SVA) is a non-enveloped, single-stranded, positive-sense RNA virus belonging to the Picornaviridae family. Senecavirus A is constantly associated with outbreaks of vesicular disease in pigs and has been reported in several countries since its first large-scale outbreak in 2014. Senecavirus A's clinical disease and lesions are indistinguishable from other vesicular foreign animal diseases (FAD). Therefore, an FAD investigation needs to be conducted for every SVA case. For this reason, SVA has been attributed as the cause of an alarming increase in the number of yearly FAD investigations performed by the United States Department of Agriculture (USDA). The objectives of this study were to estimate the seroprevalence of SVA antibodies in breeding and growing pig farms in the United States and to determine the farm-level risk factors associated with seropositivity. A total of 5,794 blood samples were collected from 98 and 95 breeding and growing pig farms in 17 states. A farm characteristics questionnaire was sent to all farms, to which 80% responded. The responses were used to conduct logistic regression analyses to assess the risk factors associated with SVA seropositivity. The estimated farm-level seroprevalences were 17.3% and 7.4% in breeding and growing pig farms, respectively. Breeding farms had 2.64 times higher odds of SVA seropositivity than growing pig farms. One key risk factor identified in breeding farms was the practice of rendering dead animal carcasses. However, the adoption of a higher number of farm biosecurity measures was associated with a protective effect against SVA seropositivity in breeding farms.

## Introduction

Senecavirus A (SVA), previously known as Seneca Valley Virus (1), is a non-enveloped, single-stranded, positive-sense RNA virus belonging to the Picornaviridae family and the only member of the genus Senecavirus (2). The virus was first isolated in 2002 as a contaminant from PER.C6 cell lines, presumably introduced via fetal bovine serum or porcine trypsin during the cell cultivation process (2). Current data suggest that SVA existed in the United States (U.S.) swine population for at least three decades (3). However, the presence of SVA in pigs with vesicular lesions had not been reported until 2007 after a trailer transporting 187 market hogs from the Canadian province of Manitoba arrived at a harvest facility in Minnesota, U.S. (4). Larger multi-state SVA outbreaks of this vesicular disease in pigs were reported in Brazil in 2014 (5), in the U.S. (6, 7), and China (8) in 2015, followed by Colombia (9), Thailand (10) in 2016, Vietnam (11) in 2018, and Mexico (12) and Chile (13) in 2022. Characteristic vesicular lesions usually start developing approximately 4 days post-infection, consisting of multiple-sized vesicles on the snout, oral cavity, and feet (i.e., coronary band and interdigital space), which may lead to lameness and lethargy. Vesicles tend to rupture 5 days post-infection (14, 15) and are clinically indistinguishable from high-consequence foreign animal diseases (FAD) such as foot-and-mouth disease (FMD). In neonatal pigs, diarrhea and a sudden increase in pre-weaning piglet mortality have also been reported (5–7), contributing to production losses.

The clinical disease and lesions caused by SVA are indistinguishable from other vesicular animal diseases: swine vesicular disease, vesicular exanthema of swine, vesicular stomatitis, and FMD. Therefore, an FAD investigation needs to be conducted by local government authorities for every SVA case. This practice, while prudent, has resulted in an increase in false alarms for FADs in the United States. The average yearly number of FAD investigations conducted by the United States Department of Agriculture (USDA) in all animal species in the U.S. between 2008 and 2014 was 487. This number increased almost four times between 2015 and 2020, with an average of 1,808 FAD investigations per year. Around 75% of FAD investigations were attributed to swine vesicular disease in pigs in the last 4 years of this period (16).

Despite the considerable number of SVA outbreak reports in swine farms across the past few years and the associated problems, the epidemiology of this disease is poorly understood. Basic information such as prevalence and risk factor studies are scarce. Therefore, the objectives of this study were to (1) estimate the seroprevalence of SVA antibodies in breeding and growing pig farms in the U.S. and (2) determine the farm-level risk factors associated with seropositivity.

## Materials and methods

The University of Minnesota (UMN) Institutional Animal Care and Use Committee (IACUC) approved this study (protocol 1804-35818A).

### Experimental design

A cross-sectional study was designed and conducted to estimate the seroprevalence of SVA in U.S. pig farms. Participation in the study was voluntary. Major veterinary clinics and production systems throughout the country were invited to participate. After agreeing to participate, each production system or veterinary clinic was asked to select breeding and growing pig farms for sample collection randomly. Both participating veterinarians and, in some cases, investigators collected the study samples.

### Sample size calculation

#### Number of farms

To calculate the number of breeding and growing pig farms to be included in the study, the following formula was used (17):


$$\begin{array}{l} N=\frac{Z^{2}pq}{L^{2}} \end{array}$$

where *N* = number of farms to be sampled, *Z* = 1.96 (*Z*-score value for 95% confidence), *p* = expected true farm-level prevalence (50% was used as the default as data was not available at the time of the study), *q* = 0.5 (1 – *p*), *L* = precision of the estimate was set at 0.1. A total of 97 breeding and 97 growing pig farms were needed for this study, which brings to a total of 194 farms.

#### Number of pigs sampled per farm

The number of samples needed within each farm to classify the farm as either seropositive or seronegative was calculated using the following formula (18):


$$\begin{array}{l} N=\frac{\log\left(1-C\right)}{\log\left(1-TP\right)} \end{array}$$

where *N* = number of animals to be sampled in each farm, *C* = 0.95 (confidence of 95%), *TP* = 0.1 (assuming that the expected true within-farm prevalence was 10%). Therefore, 29 blood samples were necessary to reach a 95% confidence level that at least one positive sample would be detected when the within-farm prevalence was at least 10%, assuming perfect sensitivity and specificity.

### Sample collection, handling, and testing

In breeding farms, sampling was performed randomly across sow parities. In the case of growing pig farms, samples were collected from 20-weeks-old or older pigs to avoid the possible detection of maternal antibodies (19). Blood samples were collected, refrigerated, and shipped to the UMN Food Centric Corridor Laboratory. Blood samples were sorted, organized, de-identified upon reception, and submitted to the UMN Veterinary Diagnostic Laboratory for testing. Senecavirus A IgG presence was tested through an immunofluorescent antibody test (IFA), which was reported to have 90% and 100% diagnostic sensitivity (Se) and specificity (Sp), respectively (20). Briefly, NCI-H1299 (ATCC^®^ CRL-5803™) cells were inoculated with SVA and fixed with cold acetone. Sera samples were screened for SVA-specific IgG in two dilutions (1:40 and 1:80) using PBS (Gibco). After incubating for 60–75 min, plates had the sera removed and washed with PBS. DyLight^®^ 650 anti-pig IgG (Abcam, Cambridge, MA) was added to the wells, and plates were incubated for 60–75 min. Plates were washed with PBS and observed under fluorescence microscopy (20) by the same laboratory technician. Fluorescence observed at a sample dilution of 1:40 or 1:80 indicated that the serum sample was positive for SVA IgG antibodies.

### Farm characteristics questionnaire

Two questionnaires—one for breeding and another for growing pig farms—were designed to capture general information such as farm type, farm size, personnel flow, animal sourcing, and other details on biosecurity measures. The survey was electronically sent to all participating veterinarians to answer on a per farm basis. Data obtained on the questionnaires were then transcribed to an electronic spreadsheet (Microsoft Excel 2016, Microsoft Corporation) for analysis.

### Data analysis

#### Estimating farm-level SVA seroprevalence

The proportions of seropositive breeding and growing pig farms were estimated after calculating the cut-point number of positive samples needed to classify a farm as being positive. The cut-point number of positive samples was determined by maximizing herd sensitivity (HSe) and herd specificity (HSp) values (21), based on the SVA IgG IFA antibody test's Se and Sp (90% and 100%, respectively) (20). Clopper-Pearson 95% confidence intervals for the proportions of seropositive farms were also calculated.

#### Association between farm type and SVA seropositivity

A chi-square test of independence was used to determine if there was a significant association between farm type (breeding or grow-finishing pig farms) and SVA seropositivity. The odds ratio and 95% CI for SVA seropositivity between farm types were calculated using the unconditional maximum likelihood estimation method (Wald).

#### Assessment of farm-level factors associated with seropositivity

The risk factor analyses for breeding and growing-pig farms were conducted separately. Univariable logistic regressions were fit to determine the unconditional associations between all risk factors recorded in the questionnaires and the outcome (SVA farm seropositivity).

Linearity between continuous variables and the outcome in the logit scale was assessed visually using scatterplots and statistically. If the relationship between the continuous variable and the outcome was not linear, continuous variables were categorized based on their median values (less than/equal to the median vs. greater than the median).

Variables with multiple categories where all positive cases were within the same category or had categories with few observations and no cases (indicating a lack of substantial variability for the analysis) were either excluded from the analysis or had their observations regrouped in a new two-factor categorical variable.

A new variable was created to evaluate the association between biosecurity measures and SVA-seropositivity. The list of biosecurity measures included in the survey was (1) Visitor check-in required to enter the farm, (2) Shower in/out procedures, (3) Danish bench-entry system is installed, (4) Use of farm-specific boots are required, (5) Use of farm-specific clothing is required, and (6) A downtime is required before entering the farm. Since all farms responded to either having or not having these six different biosecurity measures in place, they were categorized as having “four or less” or “five or six” biosecurity measures in place if they responded to having any combination of ≤ 4 or ≥5 biosecurity measures, respectively.

Unconditional associations between each predictor variable and the outcome were tabulated. Only variables with associated *p*-values below 0.2 were selected for inclusion in the multivariable analysis. A backward elimination process was used to build the final multivariable logistic model. First, a maximum model was fit using all the previously screened variables. Variables were then removed one at a time, and the likelihood ratio test was used to compare the nested models until a model with a maximum likelihood was found.

All statistical analyses were performed using R statistical software (22).

## Results

This study involved pig farms from nine production systems, eight veterinary clinics, and two private practitioners. Thirty-six swine veterinarians contributed to this study by collecting 5,794 blood samples from 193 farms. The overall survey response rate was 80% and included data from 155 (77 breeding and 78 growing pig farms) out of 193 tested farms, including all positive breeding farms and six out of the seven positive growing-pig farms.

Blood samples were collected from 193 participating farms: 98 and 95 breeding and growing pig farms located in 17 different states (Table 1). Recruitment and sample collection at all farms occurred between October 2018 and October 2019.

**Table 1:** Demographic characteristics of the 193 United States (U.S.) pig farms participating in the study.

	**Total samples**	**Breeding farms**	**Growing-pig farms**
	***n* = 193**	***n* = 98**	***n* = 95**
**Responded to survey**	155 (80%)	77 (79%)	78 (82%)
**Farm size** ^**a**^			
Range (Minimum–Maximum)	–	120–9,600	800–55,194
Median	–	2,752	3,600
Mean (S.D.)	–	3,147 (1,884)	4,922 (6,518)
**Companies** ^**b**^			
Number of participating companies	19	19	17
Median number of sampled farms per company	10	4	5
Mean number of sampled farms per company (SD)	10 (7)	5 (3)	6 (4)
**U.S. States**			
Number of participating states	17	16	11
Median number of sampled farms per Statee	5	4	6
Mean number of sampled farms per State (SD)	10 (10)	5 (5)	7 (6)

a Data from 75 and 76 participating breeding and growing-pig farms, respectively.

b Data from all participating production companies, veterinary clinics, and two private practitioners.

### Classification of farm status

The number of positive samples needed to classify a farm as seropositive was 1. This cut-off value maximized the HSp and HSe values, which reached 100% and 94%, respectively. Changing the cut-off value to 2 or 3 did not alter HSp, but HSe decreased to 77% and 51%, respectively.

### Seroprevalence results

The overall proportion of IFA-positive sera samples from breeding and growing pig sites was 4.6% (268/5,794). Of the 268 IFA-positive sera samples, 95.1% (255/268) were positive at the 1:80 dilution and 4.9% (13/268) were positive at the 1:40 dilution. Twenty-four out of 193 (12.4%) sampled farms had at least one seropositive serum sample. The median, mean, and standard deviation for the number of positive samples within positive farms were 6.5, 11.2, and 10.1, respectively.

**Figure 1:**
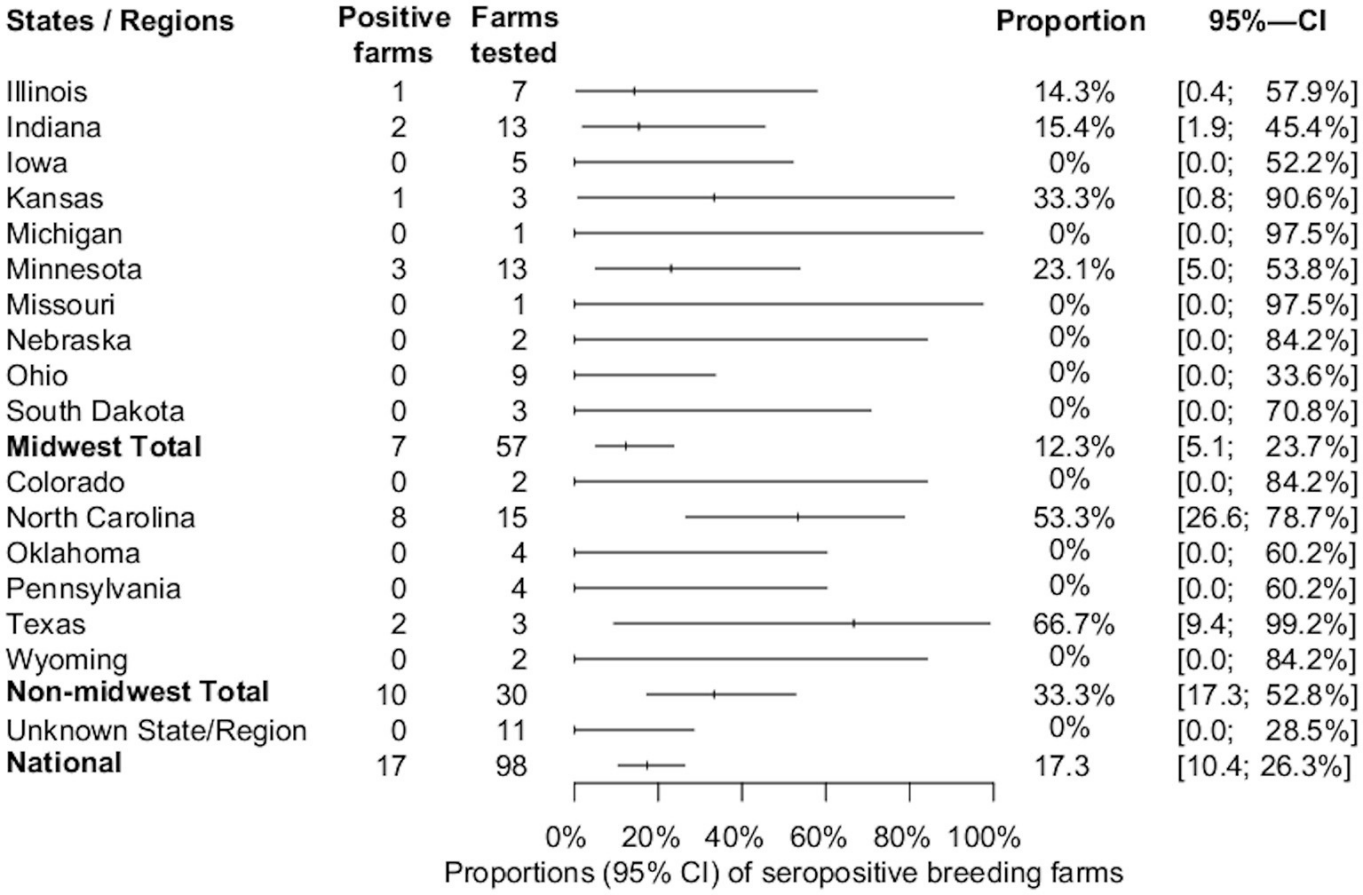
Estimated proportions and 95% confidence intervals of SVA-seropositive breeding farms by state, region, and national estimate.

**Figure 2:**
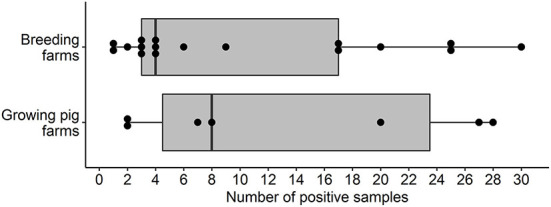
Box and whisker plot of the number of SVA IFA-positive samples by pig farm type in the U.S.

Overall, the proportion of IFA-positive sera samples from breeding farms was 5.9% (174/2,943). Seventeen out of 98 (17.3%, 95%CI: 10.4, 26.3%) breeding farms had at least one positive sample and were located in Illinois, Indiana, Kansas, Minnesota, North Carolina, and Texas. The overall estimates of SVA farm level seroprevalence among breeding farms in different states or regions are shown in Figure 1. Among seropositive breeding farms, the median, mean, and standard deviation of the number of positive samples were 4, 10.2, and 9.8, respectively (Figure 2). The average within-farm apparent prevalence among seropositive breeding farms was 34% (95% CI: 17.8, 53.5%).

**Figure 3:**
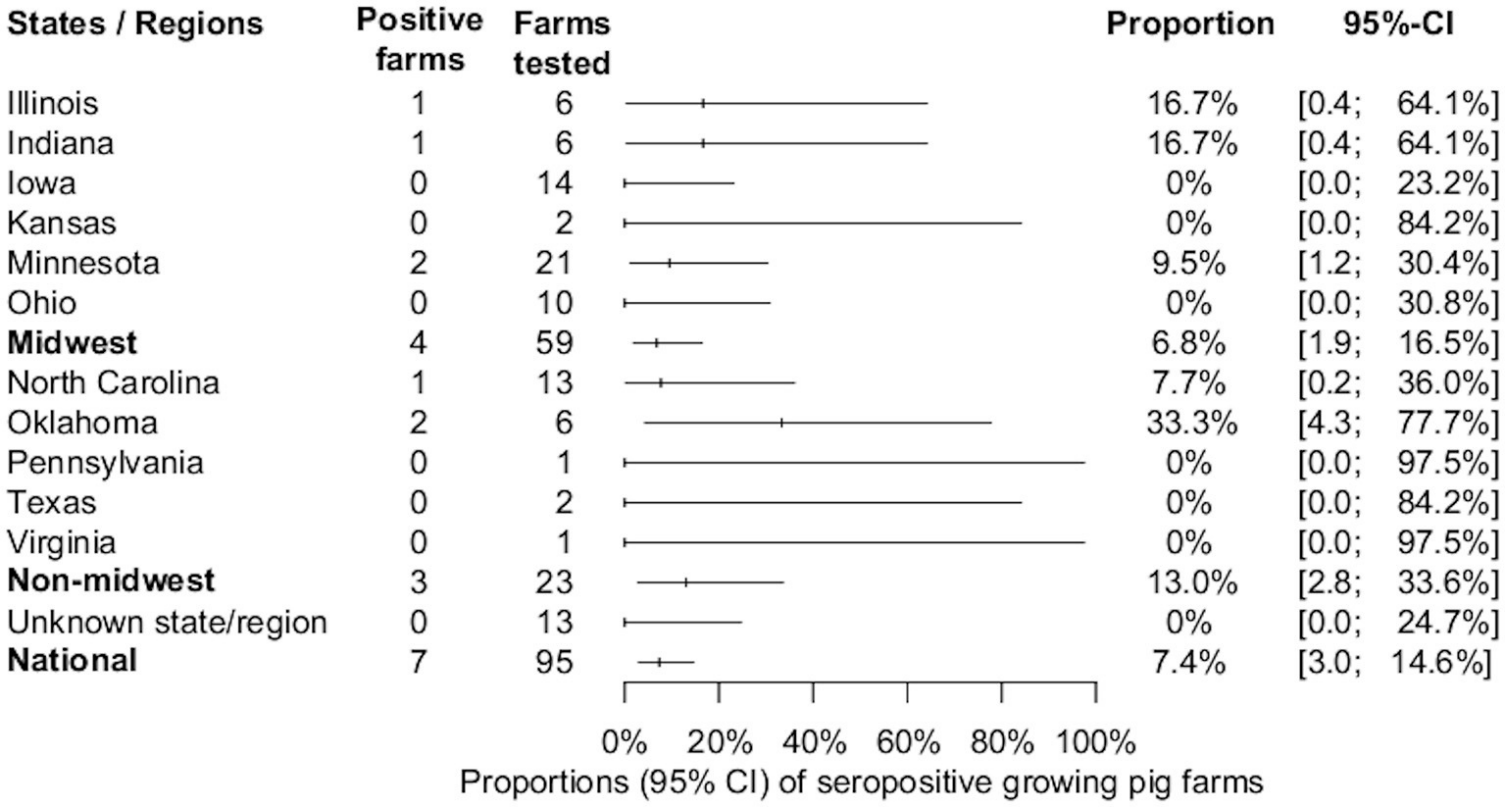
Estimated proportions and 95% confidence intervals of SVA-seropositive growing-pig farms, by state, region, and national estimate.

Seven out of 95 (7.4%, 95% CI: 3, 14.6%) growing pig farms had at least one positive sample and these were detected in the states of Illinois, Indiana, Minnesota, North Carolina, and Oklahoma. The proportion of IFA-positive sera samples from growing-pig farms was 3.3% (94/2,851). The overall estimates of SVA farm level seroprevalence among growing pig farms in different states or regions are shown in Figure 3. The median, mean, and standard deviation of the number of positive samples within positive growing pig farms were 8, 13.4, and 11.3, respectively (Figure 2). The average within-farm apparent prevalence among seropositive growing pig farms was 44.7% (95% CI: 26.6, 63.8%).

### Association between farm type and SVA seropositivity

A significant association between farm type and SVA seropositivity was detected (*X*^2^ = 4.411, *df* = 1, *p* = 0.035). Breeding farms had 2.64 (95% CI: 1.04, 6.69) times higher odds of SVA seropositivity when compared to growing pig farms.

### Risk factors associated with SVA seropositivity

After tabulation of unconditional associations between the surveyed predictors and the outcome of SVA seropositivity, six breeding and six grow-finishing pig farm predictors were selected to enter the multivariable model selection process.

The results from the univariable and multivariable logistic regression analysis for the breeding farms' characteristics are shown in Table 2. The final multivariable logistic regression model showed that breeding farms that reported rendering dead animal carcasses were more likely to be SVA-seropositive (OR = 9.2, CI: 2.5, 33.7), while farms that reported practicing five or six different biosecurity measures were less likely to be SVA-seropositive (OR = 0.2, CI: 0.1, 0.99). A summary of the biosecurity measures and associations is shown in Table 2.

**Table 2:** Univariable and multivariable logistic regression analysis of the risk factors associated with SVA seropositivity in U.S. breeding farms.

	**Univariable**			**Multivariable**		
**Characteristic**	**OR**	**95%CI**	***p*-values**	**OR**	**95%CI**	***p*-values**
**Carcass disposal method**						
Composting, burying, or incinerating	–	–	–	–	–	–
Rendering	7.9	2.4–26.7	<0.001	9.2	2.5–33.7	<0.001
Biosecurity measures in place^*^						
Four or less	–	–	–	–	–	–
Five or six	0.3	0.1–1.1	0.06	0.2	0.1–0.99	0.49
**At least one employee works on another farm**						
No	–	–	–	–	–	–
Yes	0.2	0.0–1.7	0.1	–	–	–
**Type of manure storage**						
Uncovered lagoon	–	–	–	–	–	–
Deep pits	0.2	0.1–0.8	0.02	–	–	–
**Water treatment**						
No	–	–	–	–	–	–
Yes	0.2	0.0–0.9	0.03	–	–	–
**Cull sows and weaned piglets use the same ramp when truck loading**						
No	–	–	–	–	–	–
Yes	0.4	0.1–1.4	0.16	–	–	–

* Farms were categorized as having “four or less” or “five or six” biosecurity measures in place if they responded to having any combination of ≤ 4 or ≥5 biosecurity measures in place, respectively, from a list of six biosecurity measures included in the survey.

It was not possible to fit a multivariable logistic regression model for the growing-pig farms due to the low number of seropositive farms. The risk factors in growing-pig farms that appeared to be positively associated with SVA-seropositivity (*p* < 0.2) in the univariable analysis were (1) pigs are loaded into trucks by an external pig-loading crew, and (2) More than one external crew is hired to perform jobs at the farm. Alternatively, the risk factors that appeared to be negatively associated with SVA-seropositivity (*p* < 0.2) were (1) people that load pigs into trucks have direct access to pigs in the barn after loading a truck, (2) all pigs in the farm are sourced by a single breeding farm, and (3) all trucks that arrive in the farm are cleaned and disinfected (Table 3).

**Table 3:** Univariable logistic regression analysis of the risk factors associated with SVA seropositivity in U.S. growing pig farms.

	**Univariable**		
**Characteristic**	**OR**	**95%CI**	***p*-values**
**External pig-loading crew** ^**a**^			
No	–	–	–
Yes	9.3	1.03–84.9	0.047
**Direct access to pigs in the barn after loading a truck** ^**b**^			
No	–	–	–
Yes	0.1	0.01–0.8	0.035
**Hires more than one external crew** ^**c**^			
No	–	–	–
Yes	3.3	0.6–19.2	0.188
**Single-sourced pigs** ^**d**^			
No	–	–	–
Yes	0.3	0.05–1.6	0.158
**All trucks come clean and disinfected** ^**e**^			
No	–	–	–
Yes	0.3	0.05–1.7	0.165

a Market pigs are loaded into trucks by an external pig-loading crew.

b People who load pigs into trucks have direct access to pigs in the barn after loading a truck.

c More than one external crew is hired to work at the farm.

d All pigs on the farm are sourced from a single breeding farm.

e All trucks that arrive on the farm are cleaned and disinfected.

## Discussion

The present study demonstrates that SVA antibodies exist in the U.S. swine population. To the authors' knowledge, this is the first national study designed and sampled to estimate the seroprevalence of SVA. Despite the high incidence of swine-vesicular FAD investigations (16), the estimated farm level apparent seroprevalences of 17.3% (95%CI: 10.4, 26.3%) and 7.4% (95% CI: 3, 14.6%) among U.S. breeding and growing pig farms, respectively, were relatively low. These proportions change slightly when accounting for the imperfect HSe estimate of the applied methodology. Considering the calculated HSe (94%) and HSp (100%), breeding and growing pig farms had estimated true seroprevalences of 18.5% (95%CI: 11.1, 28%) and 7.8% (95%CI: 3.2, 15.5%). Although slight numerical increases are seen when comparing the apparent and true prevalence estimates, there are no significant changes due to the overlapping confidence intervals.

Currently, there is scarce information on the serological response to SVA at a population level. In a recent study, SVA IgG was detected in a cohort of 60 sows from a 6,000-sow farrow-to-wean farm that underwent an SVA outbreak for up to 13 months after the outbreak, using the same IFA procedure (23). This suggests that antibodies can be detected for an extended period after exposure. Therefore, the IgG detection in this study is likely the result of naturally-infected breeding and growing pig farms, even if exposure happened a long time before sampling, as maternally derived antibodies may be undetectable after 6 weeks of age (19).

It is currently unknown what may have caused the onset of large-scale SVA outbreaks after 2014–2015. The virus is likely to have been circulating within and between U.S. pig farms since at least 1988, as was shown by the sequence analysis of picorna-like viruses isolated from pigs in the U.S. (3). Conversely, another retrospective study attempted to assess the presence of SVA in Brazil through the serological testing of samples collected between 2007 and 2016 (24). The authors concluded that SVA was likely absent in the major Brazilian pig-producing states before 2014. However, the reported results must be interpreted carefully since a low number of samples were tested and collected from asymptomatic farms for other research purposes not related to vesicular diseases. Therefore, the study design likely introduced a selection bias that significantly reduced the probability of detecting SVA-exposed animals. A more comprehensive study design is needed to rule out the presence of SVA among Brazilian pig farms before 2014. It may be possible that SVA can remain present and undetected in pig populations until a formal vesicular disease investigation is conducted and SVA is ruled out.

The results from this study differ significantly from the results of another seroprevalence study conducted in U.S. pig farms using samples collected in 2016 (25). The estimated farm-level seroprevalences were 75.8% in breeding farms and 42.7% in growing pig farms vs. 17.3% and 7.4% in this study. The discrepancies in the proportions reported in both studies may be explained by fundamental differences in the study designs, time periods when samples were collected, and interpretation of the serological assays. This study's source population was U.S. pig farms from major swine-producing companies and veterinary clinics, regardless of their SVA or other infectious diseases status. However, in the study by Houston et al. (25), the source population was pig farms conducting porcine reproductive and respiratory syndrome virus (PRRSv) monitoring at one Veterinary Diagnostic Laboratory with no known history of SVA. It is currently unknown if the presence of other infectious diseases (e.g., PRRS) could be associated with the presence of SVA, which may have introduced potential biases. It is possible that biosecurity failures in PRRSv-positive farms are also responsible for the introduction of SVA; thus, assessing the prevalence of SVA exposure exclusively in farms monitoring for PRRSv is not appropriate. The parallel interpretation of two different serological tests with fair-to-moderate results agreement by Houston et al. (25) may have overestimated the proportion of positive farms, partially explaining the significant differences between both studies.

Very little is known about how SVA transmits between farms. Senecavirus A-infected animals appear to develop a short-term viremia for up to 10 days post-infection, and shed the virus for up to 28 days post-infection in oral/nasal secretions and feces (14). While this information can help us mitigate transmission between animals on a farm, more information is needed to prevent the infection of pig farms in the first place. To shed some light on this matter, we performed a risk-factor analysis to identify what farm characteristics might be associated with SVA exposure.

Implementation of biosecurity measures in breeding farms yielded a sparing effect in this study. While not surprising, it does remind the industry of the importance and needs that most modern pig farms have when adopting such preventive measures to avoid the introduction of new pathogens carried by people themselves or the boots and clothes they are wearing (26). However, rendering was another predictor in the model that was found to have a significant association with SVA seropositivity. Breeding farms that reported disposing of the carcasses of dead animals via rendering had 9.2 higher odds of being seropositive compared to farms that either compost, bury, or incinerate the dead animals. One possible causal pathway for this association is the indirect transmission of the pathogen between farms through the trucks transporting the carcasses since the truck may need to visit several farms before filling and returning to the rendering plant. Similar associations involving the disposal of dead animals via rendering have been reported in other studies, such as with the increased risk of respiratory disease outbreaks in pig farms (27), PRRSv positivity (28), porcine epidemic diarrhea positivity (Morrison Swine Health Monitoring Project science page, personal communication), H5N2 highly pathogenic avian influenza virus (29), and H7N2 avian influenza virus in commercial poultry farms in the United States (30). More studies are needed to understand whether other carcass disposal methods should be considered to reduce the probability of introducing SVA or any other pathogen to the farm.

Due to the low number of positive observations, it was not possible to build a multivariable logistic regression model for the growing pig farms. The positive univariable associations between “external pig loading crew” and “hires more than one external crew” with the outcome of seropositivity (Table 3) highlight the potential role of people in the introduction of pathogens. As for the protective associations, it is not surprising that single-sourcing of weaned pigs and disinfecting all incoming trucks would decrease the odds of seropositivity since such measures prevent the comingling of pigs from negative and positive populations and the cross-contamination between different batches of animals, respectively. However, the statistically significant association of being at lesser odds of positivity when people had direct contact with pigs in the barn after loading pig trucks is unexpected and challenging to explain. Upon further investigating this artifact of the analysis, it was seen that the only farms where people went back into the barns and had contact with the remaining pigs were the ones that did not hire an external pig-loading crew. All farms that hired an external pig-loading crew reported that people left the farms after loading the trucks without contacting the remaining pigs. Therefore, it is likely that this association is measuring a similar effect as the association with the farms that hire external pig-loading crews.

Although the current study design is not optimal for estimating within-farm prevalences, a broad range of SVA-positive sera samples was detected. The range of positive samples per positive farm was somewhat similar between the breeding and growing pig farms (Figure 2), with an average of 10.2 and 13.4 out of 30 tested samples in breeding and growing pig farms, respectively (Figure 2). As reported in the results section, the estimated within-farm prevalence yielded wide confidence intervals due to the reduced sample size per farm. However, these results may still provide helpful information for further investigations. Nevertheless, interpreting these results is rather difficult in cross-sectional studies since there is no information about the previous SVA history on tested farms.

## Conclusion

This is the first study specifically designed to estimate the seroprevalence of SVA at a national level, with a broad selection of farms from producing companies and veterinary clinics as the source population. After sampling and testing 5,794 sera samples from 98 breeding and 95 growing pig farms, it was shown that SVA antibodies are present among U.S. pig farms. Seroprevalence was higher in breeding farms than in growing pig sites.

Key risk factors identified were the rendering of dead animals and access of external working crews to the farms. At the same time, the implementation of biosecurity measures seemed to have a protective effect against SVA seropositivity. These findings may be applied in pig farms to help reduce the risk of SVA exposure. Other carcass disposal methods could be considered, such as composting or incineration, or the dead-animal disposal areas should be located away from the farms, and the trucks used for carcass collection should be prohibited from coming close to the barns. Furthermore, attention should be given to biosecurity measures to reduce the risk of pathogen introduction through any incoming personnel or fomites. Although this is the first assessment of farm-level risk factors associated with SVA seropositivity, more studies need to be specifically designed to understand these associations.

## Data availability statement

The raw data supporting the conclusions of this article will be made available by the authors upon reasonable request.

## Ethics statement

The animal study was reviewed and approved by Institutional Animal Care and Use Committee, University of Minnesota, Minneapolis, United States. Written informed consent was obtained from the owners for the participation of their animals in this study.

## Author contributions

JS, CV, and CC wrote the research proposal. GP and CC developed the farm-characteristics questionnaire and contacted the veterinarians for sample collection and questionnaire response. MC helped with the questionnaire preparation. GP compiled all test results and questionnaire responses and analyzed the data with JS and CC. FV contributed to the diagnostic data interpretation. GP wrote the initial draft of the manuscript. All authors read, edited, and approved the finalized manuscript.

## Funding

This research was funded by the National Pork Board (NPB#18-041).

## Conflict of interest

The authors declare that the research was conducted in the absence of any commercial or financial relationships that could be construed as a potential conflict of interest. The handling editor EM declared a shared affiliation with the author CV at the time of review.

## Publisher's note

All claims expressed in this article are solely those of the authors and do not necessarily represent those of their affiliated organizations, or those of the publisher, the editors and the reviewers. Any product that may be evaluated in this article, or claim that may be made by its manufacturer, is not guaranteed or endorsed by the publisher.
